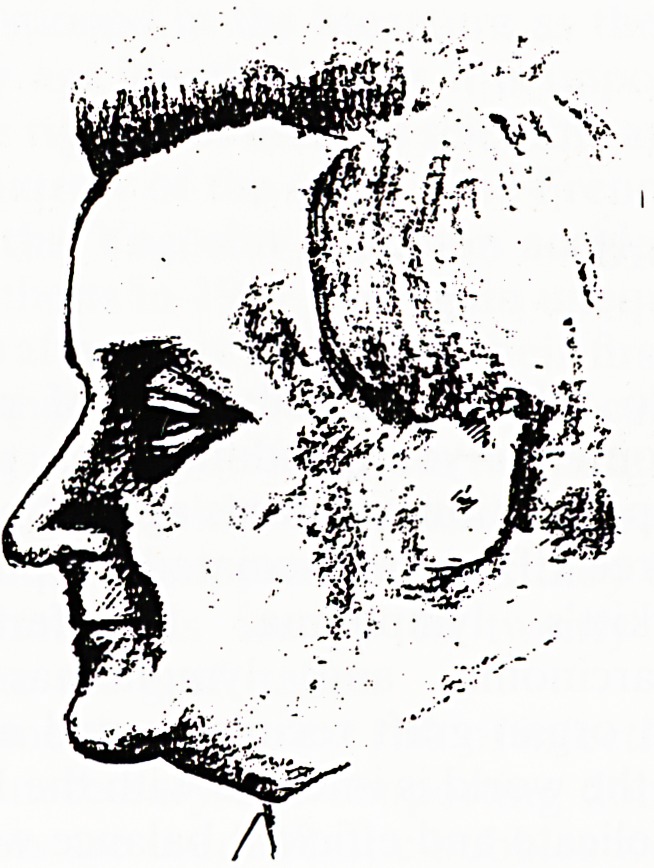# Molecular Medicine

**Published:** 1988-11

**Authors:** D. J. Weatherall

**Affiliations:** Nuffield Department of Clinical Medicine, John Radcliffe Hospital, Oxford


### Molecular Medicine

**Professor Sir D. J. Weatherall**
Nuffield Department of Clinical Medicine, John Radcliffe Hospital, Oxford

Professor Weatherall began by saying that he was very aware of the dangers of trying to cover an enormous subject in half an hour but he would try and give us a bird's eye view of molecular medicine, and cone it down on one or two areas in a little bit more detail. He started with the proposition that in the next 25 years there would be a change in medical research which will take investigation from 'whole patient physiology' to the analysis of diseases at the cellular and molecular level.

In the mid-1970s some quite extraordinary things happened, techniques became available which made it possible to look at human genes in detail. The first was the hybridisation of DNA. The next was the family of enzymes that cut up DNA–restriction enzymes. There are several hundred enzymes which will chop up DNA and give exactly the same sized pieces every time. These pieces can be inserted into plasmids or bacteriophage, or a variety of so-called vectors, to make recombinant molecules, these can be put into bacterial cells to grow up the pieces of DNA and clone them. Next, extraordinary techniques were developed for sequencing and mapping DNA. Then techniques were developed for making tiny little bits of DNA, augmenting them in the test tube over a few hours, so that from the diagnostic point of view one has a completely new tool. Finally came the idea that one can take foreign DNA, transfer it into a foreign system, into another cell or into another animal, look at its expression and learn how genes are regulated. So those are the tools of the trade and they all happened very quickly and in clinical practice they have a spectrum of uses.

The great excitement is to define the genes which are involved in making the individual genetically susceptible to diseases, to ask what those genes do and what are their products, thereby learning something about the aetiology of these conditions.

The first single gene disorder to be looked at at the molecular level was thalassaemia – severe genetic anaemia. The basic problem is haemoglobin synthesis and difficulty in making beta chains, and the question is what is the matter with the gene which regulates the structure of the beta chains of haemoglobin in these children? Now it is possible to take the genes out from these children, clone and then sequence them. Studying this disease has told us perhaps the whole repertoire of things that can go wrong with genes.

It is now apparent that over 50 different mutations can cause the clinical picture of thalassaemia. Sometimes the genes are missing (deleted) but in the majority of cases there are simple basic changes in the DNA which cause inefficient transcription or translation. For example base changes in the coding regions (exons) may scramble the genetic code and lead to the production of a useless messenger RNA. Similarly, base changes in the critical regions near the junctions between introns and exons may prevent the normal splicing reactions which join the exons together and form the definitive messenger RNA. Other more subtle mutations may involve regulatory regions outside the gene. As other genetic diseases are studied in this way similar lesions are turning up. For example haemophilia is very heterogenous; some cases are due to deletions of the factor VIII gene while others are due to mutations that scramble the genetic code. It is now possible to use this information to develop prenatal diagnosis programmes using fetal DNA obtained by chorionic villus in the first trimester.

Another major success story in this field is the development of methods for finding genes for diseases, the cause of which is completely unknown. This has entailed linkage studies using DNA polymorphisms, i.e. normal variation in the structure of DNA that produces a new site for a cutting enzyme. For example it has been possible to find the gene for Duchenne muscular dystrophy on the X chromasome. Once having found a gene it is possible, from its sequence, to work back to its protein product and hence to find out what is the basis of the disease. In the case of the Duchenne muscular dystrophy it is the defective production of a muscle protein called dystrophin.

In the future it should be possible to use a similar linkage approach to define some of the genes that are involved in the genetic susceptibility to diseases like coronary artery disease and major psychiatric illness. Recent studies on the pathogenesis of cancer have also been facilitated by the molecular approach in that it is now possible to define many cancers in terms of one or more mutations of specific cellular housekeeping genes called oncogenes.

Recombinant DNA technology is also allowing us to manufacture a variety of pharmaceutical products by putting human genes into bacteria and having them express themselves in their new home. Genetically engineered erythropoietin for the treatment of the anaemia of renal disease is a good example; many will follow. Recombinant DNA technology is also allowing us to produce a wide range of diagnostic agents for the rapid identification of bacteria, viruses and parasites. In the long term it should help us develop vaccines for many of these conditions.

Clearly molecular medicine is here to stay and has wide application right across clinical medicine. Indeed clinical genetics can be defined as 'anything interesting', a term first used by Sir Cyril Clarke many years ago and certainly more true than ever today.

Sir David ended with the thought that the most important benefits of all from the biotechnology field in the next 100 years would come from growing better plants.

*In reply to questions –*

Dr Mott asked him to speculate on the development of new vaccines. Sir David discussed the efforts to develop a vaccine against malaria, various phases have been gone through; first identification of likely antigens which might be protective and then making large quantities by cloning them. This initial approach has not been very successful so far. The next stage may be to find out which part of the antigen is the 'business bit' e.g. small peptides and to use those as immunogens. The yeast cloning of antigens has been very successful against hepatitis. It may go the same way with malaria.

Dr Laszlo asked about the need to regulate research into gene therapy. Sir David said that he had just been chairing a committee at the MRC to get some guidelines for gene therapy in human beings. The committee has said that scientists should not be allowed for the forseeable future to put genes into fertilised human eggs for replacement – genes that would subsequently be expressed in future generations. He mentioned the government's white paper on embryo research and he hoped that the Bill would go through as it stands. He said that society must be able to feel that these matters were being looked at carefully. He did not think that scientists could have total freedom in this field, if they do not act responsibly the public will just come down on them and they will end up with a situation in which they can do nothing.

Dr Payne asked him to look into the future and tell us what the difference in life expectation might be in a few hundred years time. Sir David said that it was likely that the known hazards to life from malignant and degenerative disease would be removed and then the question would be would it ever be possible to prolong the ageing process ? Ageing must be genetic because different species have their own very tight kind of natural age. How this works is fascinating, if we could discover which genes are at work in cell ageing we might be able to modify them. It is conceivable that ultimately we may be able to do this and to live a lot longer.

## Figures and Tables

**Figure f1:**